# Pro-inflammatory Ca^++^-activated K^+^ channels are inhibited by hydroxychloroquine

**DOI:** 10.1038/s41598-017-01836-8

**Published:** 2017-05-15

**Authors:** María Eugenia Schroeder, Sofía Russo, Carlos Costa, Juliana Hori, Inés Tiscornia, Mariela Bollati-Fogolín, Darío S Zamboni, Gonzalo Ferreira, Ernesto Cairoli, Marcelo Hill

**Affiliations:** 1 Laboratory of Immunoregulation and Inflammation, Institut Pasteur de Montevideo, Montevideo, Uruguay; 2 Immunobiology Department, Montevideo Faculty of Medicine, University of the Republic, Montevideo, Uruguay; 3 Ion Channels Laboratory, Biophysics Department, Montevideo Faculty of Medicine, University of the Republic, Montevideo, Uruguay; 4 Department of Cell Biology, School of Medicine of Ribeirão Preto, University of São Paulo, FMRP/USP, Ribeirão Preto, São Paulo Brazil; 5 Laboratorio de Biotecnología, Facultad de Ingeniería-Universidad ORT Uruguay, Cuareim 1451, 11100 Montevideo, Uruguay; 6 Unit of Systemic Autoimmune Diseases, “C” Medical Clinic Prof. Dr. Juan Alonso Bao, Hospital of Clinics, Montevideo Faculty of Medicine, University of the Republic, Montevideo, Uruguay

## Abstract

Antimalarials have demonstrated beneficial effects in Systemic Lupus Erithematosus and Rheumatoid Arthritis. However, the mechanisms and the molecular players targeted by these drugs remain obscure. Although hydroxychloroquine (HCQ) is a known ion channel inhibitor, this property has not been linked to its anti-inflammatory effects. We aimed to study whether HCQ inhibits pro-inflammatory ion channels. Electrophysiology experiments demonstrated that HCQ inhibited Ca^++^-activated K^+^ conductance in THP-1 macrophages in a dose-dependent manner. In macrophages, ATP-induced K^+^ efflux plays a key role in activating the NLRP3 inflammasome. ATP-induced IL-1beta secretion was controlled by the KCa1.1 inhibitor iberiotoxin. NS1619 and NS309 (KCa1.1 and KCa3.1 activators respectively) induced the secretion of IL-1beta. This effect was inhibited by HCQ and also by iberiotoxin and clotrimazol (KCa3.1 inhibitor), arguing against off-target effect. *In vitro*, HCQ inhibited IL-1beta and caspase 1 activation induced by ATP in a dose-dependent manner. HCQ impaired K^+^ efflux induced by ATP. *In vivo*, HCQ inhibited caspase 1-dependent ATP-induced neutrophil recruitment. Our results show that HCQ inhibits Ca^++^-activated K^+^ channels. This effect may lead to impaired inflammasome activation. These results are the basis for i) a novel anti-inflammatory mechanism for HCQ and ii) a new strategy to target pro-rheumatic Ca^++^-activated K^+^ channels.

## Introduction

Antimalarials have demonstrated beneficial effects in Systemic Lupus Erithematosus (SLE) and Rheumatoid Arthritis (RA). In SLE management, hydroxychloroquine (HCQ) is considered as the “gold standard” therapy^1^. However, the mechanisms by which HCQ helps to control pathogenic inflammation as well as molecular targets controlled by the drug are poorly understood^2^. Additional knowledge in this field may certainly help to rationalize its clinical use. HCQ is a derivative of quinine, which is a well-known K^+^ channel inhibitor. HCQ has also been described to inhibit ion channels^3^. Ion channels are key regulators of innate and adaptive immune responses^4^. However, to our knowledge, the anti-inflammatory properties of HCQ have not been attributed to its ability to block ion channels. We therefore aimed to study whether HCQ inhibits pro-inflammatory ion channels.

Among others, the Ca^++^-activated K^+^ channels KCa1.1 and KCa3.1 have been involved in autoimmunity as well as cardiovascular disease^5–9^. KCa1.1 (SLO1, BKCa) are high conductance K^+^ channels activated both by membrane depolarization and cytosolic Ca^++^. KCa1.1 are homotetramers of 4 pore-forming α-subunits, whose topology resembles that of voltage-gated K^+^ (K_V_) channels. The C-terminus appears to confer ion sensitivity since Ca^++^ binding sites and regulatory domains have been identified in this region of the KCa1.1 α subunit. In some but not all tissues, auxiliary β-subunits interact with KCa1.1 α-subunits to provide functional diversity. The intermediate-conductance Ca^++^-activated K^+^ channel is composed of 4 KCa3.1 subunits and 4 calmodulin molecules. Unlike KCa1.1, KCa3.1 activation is voltage independent.

KCa1.1 channels are expressed in fibroblast-like synoviocytes (FLS) from Rheumatoid Arthritis patients^9^ and from rats with pristane-induced arthritis^7^. Its pharmacologic inhibition controls FLS proliferation, invasion as well as secretion of pro-inflammatory mediators^8, 9^. As for KCa3.1, this channel is expressed by T cells^10–13^, macrophahes^5^ as well as DCs^14^. It has recently been reported that KCa3.1^−/−^ mice do not develop collagen-induced arthritis^6^. In agreement with this, KCa3.1 inhibition leads to controlled proliferation and secretion of pro-inflammatory molecules in RA FLS^7^. KCa1.1 and KCa3.1 have therefore been proposed as a molecular targets to control RA^4^.

One of the processes in which pro-inflammatory ion channels play a role is the activation of inflammasomes. Inflammasomes are cytosolic multiprotein complexes formed by a receptor, an adaptor molecule and caspase 1^15^. Canonical inflammasome assembly leads to the activation of pro-caspase 1 that then proteolitically activates the pro-inflammatory cytokines IL-1beta and IL-18. Inflammasomes are believed to play a role in lupus predisposition and pathogenesis^16, 17^ as well as in RA^18, 19^. Intracellular Ca^++^ and K^+^ levels are believed to control inflammasome activation, although the role played by Ca^++^ is a matter of debate in this field^15^.

Since the ion channel inhibitor HCQ is a widely used anti-rheumatic drug and Ca^++^-activated K^+^ channels promote autoimmunity, we speculated that HCQ may inhibit those conductances. Here we show that HCQ inhibits Ca^++^-activated K^+^ channels probably leading to impaired inflammasome activation. These results are the basis for i) a novel anti-inflammatory mechanism for HCQ and ii) a new strategy to target pro-rheumatic Ca^++^-activated K^+^ channels.

## Results

**Figure 1:**
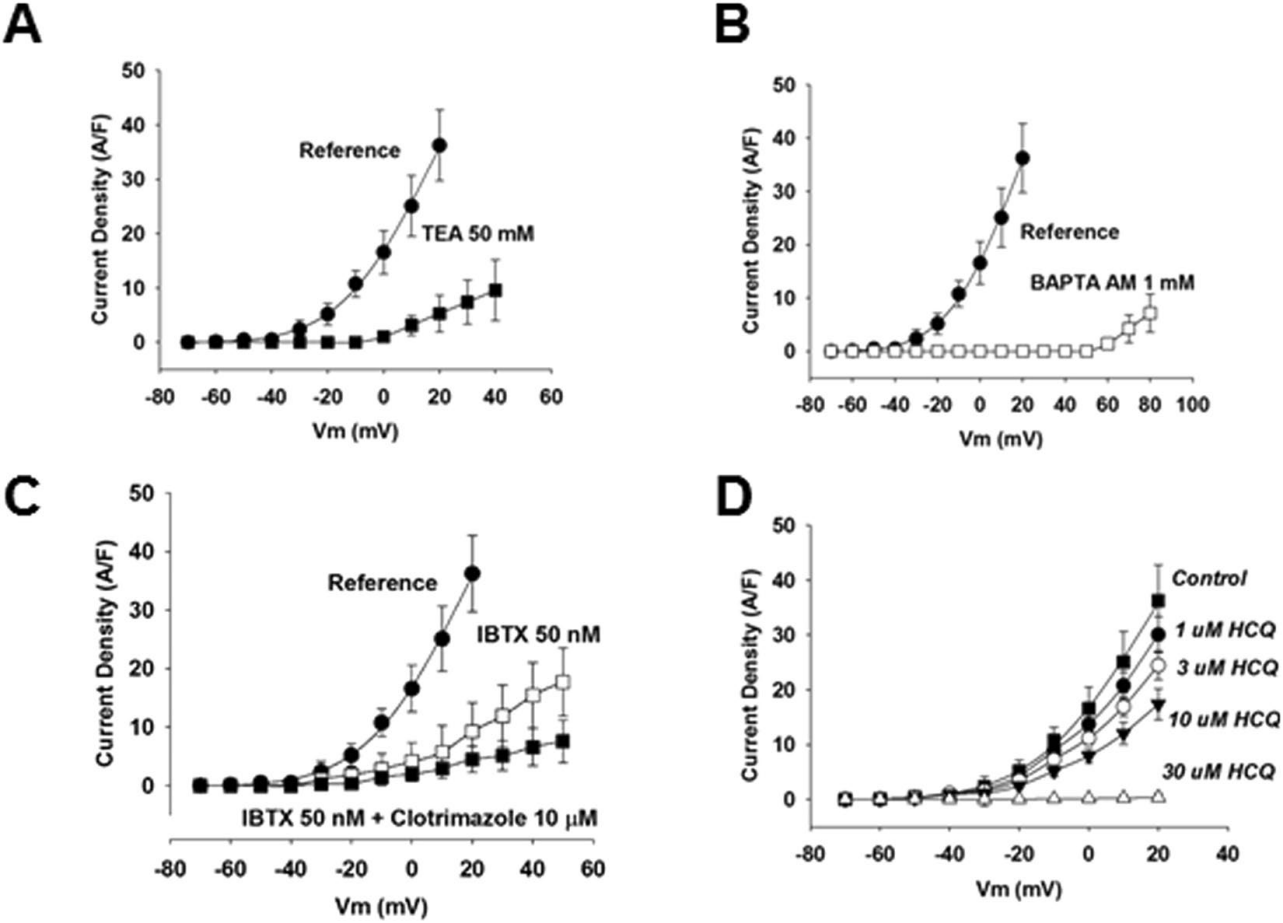
HCQ inhibits Ca^++^-activated K^+^ channels. Electrophysiology registers expressed as current density obtained through the whole cell path clamp technique using THP-1 cells. TEA is a K^+^ channel inhibitor, BAPTA AM is an intracellular Ca^++^ chelator. Symbols are averaged normalized currents ± SEM (n = 5, p < 0.05).

We hypothesized that HCQ may inhibit the pro-rheumatic Ca^++^-activated K^+^ channels. To directly address this question, we performed electrophysiology experiments in THP-1 macrophages. THP-1 macrophages are known to express Ca^++^-activated K^+^ channels KCa1.1 and KCa3.1^20^. We were able to record outward currents triggered by depolarizations starting at −20 mV and that were inhibited by TEA, BAPTA AM and IBTX (Fig. 1A–C) getting rid of other likely K^+^ conductances as described in “Methods”. When adding 10 uM clotrimazole to the extracellular solution containing IBTX 50 nM, a very small current (about 5% of the total current) remained, especially at positive potentials (Fig. 1C). Thus, most of the calcium-activated potassium conductance was blocked by the combination of IBTX and clotrimazole. These results strongly suggest that calcium-activated potassium conductance is almost exclusively mediated by KCa3.1 and KCa1.1 in THP-1 macrophages. We then studied whether these currents could be inhibited by HCQ. In agreement with our hypothesis, we observed a dose-dependent inhibition which was maximal at 30 µM (Fig. 1D).

**Figure 2:**
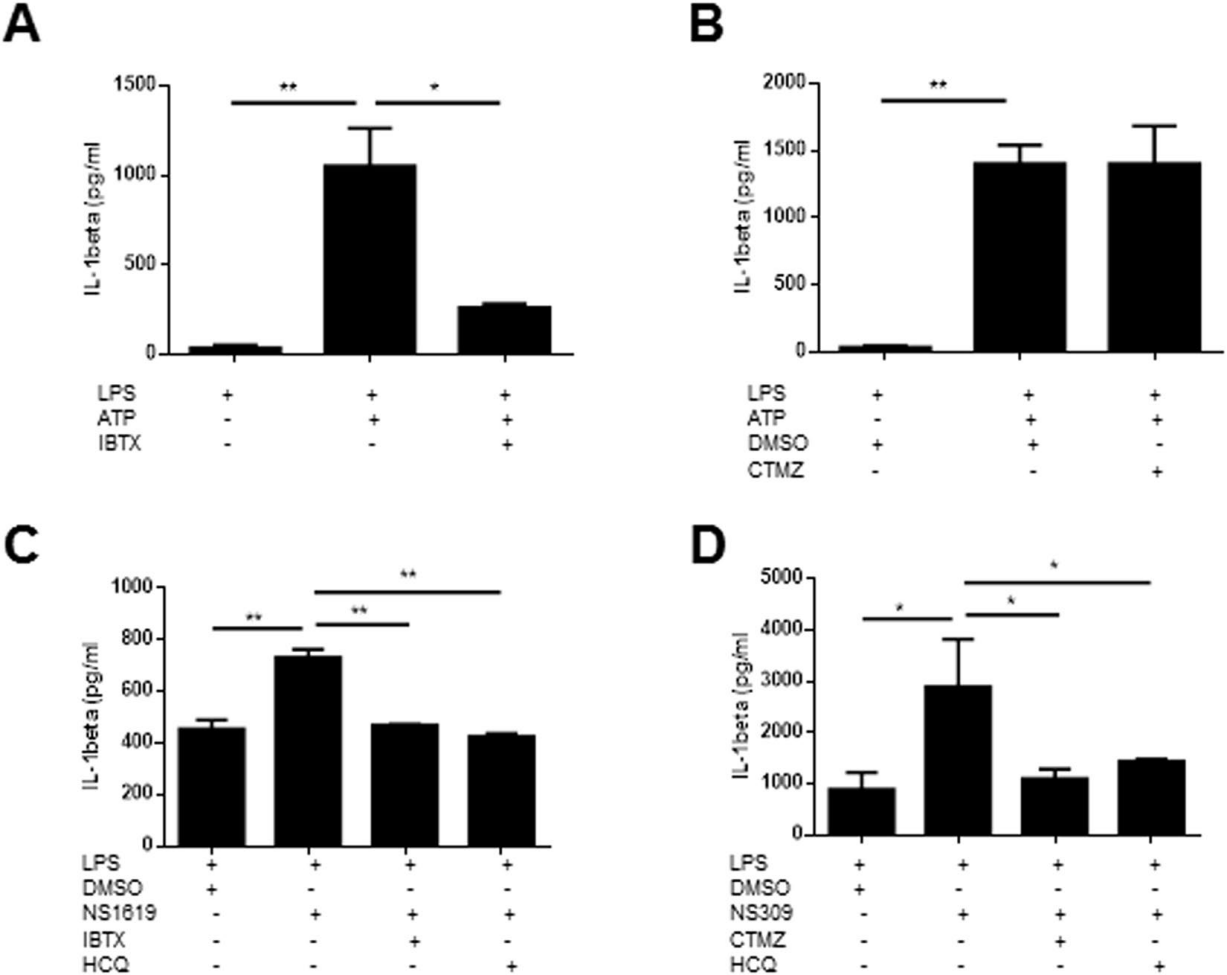
KCa1.1 and KCa3.1 may be involved in inflammasome activation. IL-1beta secretion by THP-1 cells under the indicated treatments. NS1619 (25 µM) and Ibtx (50 nM) are KCa1.1 activator and inhibitor respectively. NS309 (10 µM) and clotrimazole (10 µM) are KCa3.1 activator and inhibitor respectively. DMSO was used as vehicle control. One experiment representative of three is shown. *p < 0.05; **p < 0.01; ***p < 0.001.

We then wished to understand the mechanisms by which these channels may have pro-inflammatoy properties in human macrophages that would be relevant in rheumatic diseases. It is well known that K^+^ efflux plays a key role in ATP/P2X7R-induced inflammasome activation. Although a role for cytosolic Ca^++^ has been proposed in this pathway, its importance is a matter of debate. To study this issue we treated LPS-primed THP-1 cells with ATP and quantified IL-1beta by ELISA. We modulated KCa1.1 and KCa3.1 with specific pharmacologic activators and inhibitors. Interestingly, IL-1beta secretion induced by ATP was controlled by a KCa1.1 inhibitor like IBTX at 50 nM (Fig. 2A). However, ATP-induced IL-1beta secretion was not inhibited by a KCa3.1 inhibitor (Fig. 2B). KCa1.1 is known to be activated by depolarization and Ca^++^, whereas KCa3.1 is voltage-independent^4^. Thus, the Ca^++^ increase triggered by ATP might not be enough to activate KCa3.1. However, Ca^++^ increase in the context of strong depolarization triggered by ATP may activate KCa1.1. We then activated KCa1.1 and KCa3.1 by using NS1619 and NS309, respectively. Interestingly, both drugs separately induced IL-1beta secretion in the absence of ATP (Fig. 2C,D). These observations are unlikely due to off-target effects since they were blocked by the inhibitors iberiotoxin (KCa1.1) and clotrimazole (KCa3.1) (Fig. 2C,D). Importantly, HCQ was also able to inhibit NS1619 and NS309-induced IL-1beta secretion. (Fig. 2C,D). Thus, our results suggest that KCa1.1 and KCa3.1 may promote inflammasome activation. Moreover, our results also suggest that HCQ could be an inflammasome inhibitor. We were then interested in studying this hypothesis.

**Figure 3:**
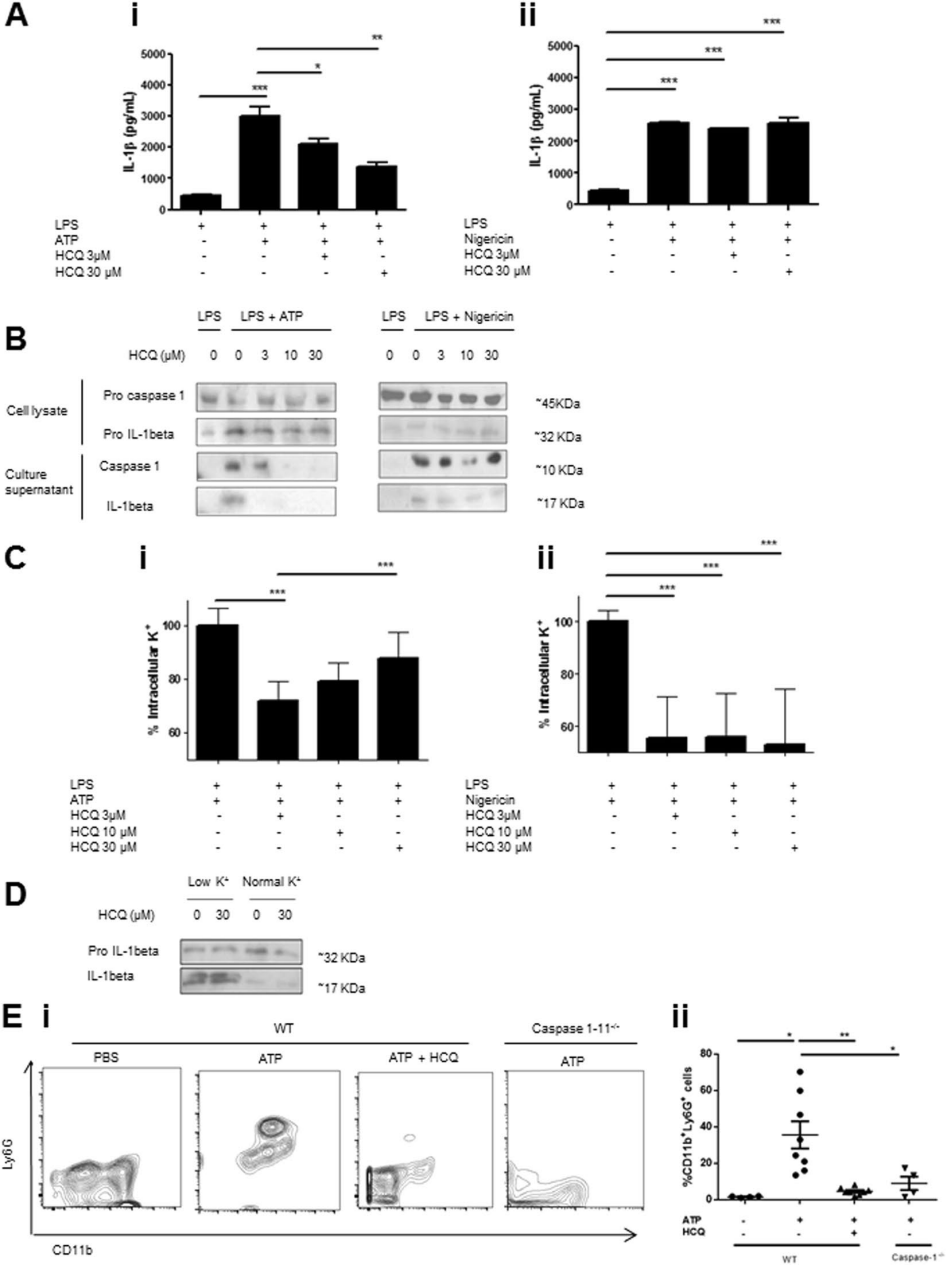
HCQ inhibits ATP-induced inflammasome activation. *In vitro* experiments were carried out with PMA-differentiated THP-1 macrophages. (**A**) Cells were primed with 0.25 µM LPS for 3 hours and then washed. When HCQ was used, it was added at this step during 15 minutes. Without further washing, cells were then treated for 45 minutes with 5 mM ATP or 2.5 µM nigericin, in the presence or not of HCQ. Culture supernatant was harvested and IL-1beta was quantified by ELISA. One experiment representative of five is shown. (**B**) Western blot analyses of culture supernatant of THP-1 cells treated with the indicated compounds. One experiment representative of five is shown. (**C**) K^+^ efflux was studied by quantifying intracellular K by spectroscopy under the indicated treatments. A pool of three independent experiments is shown. (**D**) Western blots studying IL-1beta maturation in cells cultured during 45 minutes with physiological buffer or without K^+^. One experiment representative of two is shown. (**E**) WT or caspase-1^−/−^ C57Bl/6 mice were injected with 20 mg/kg ATP. 4 hs later, peritoneal lavage was performed and cells were stained with anti-CD11b and Ly6G antibodies. In HCQ-treated animals, 1 mg/kg of the drug was injected i/p daily 7 days before ATP injection. i) Representative dot plots are shown. ii) Neutrophil quantification from two independent experiments. *p < 0.05; **p < 0.01; ***p < 0.001.

We cultured human and mouse macrophages and dendritic cells (DCs) in which NLRP3 inflammasome was activated by ATP or nigericin, in the presence or not of HCQ. ATP is known to act on P2X7 ionotropic receptors leading to K^+^ and Ca^++^ mobilization and inflammasome activation. In contrast to particulate stimuli, ATP does not induce the acidification of endolysosomes^21^. Thus, a potential effect of HCQ in this system should not be explained by its lysosomotropic activity. Nigericin is an ionophore that mediates K^+^ efflux. We observed that HCQ inhibited ATP-induced IL-1beta secretion in a dose-dependent manner in all the cell types studied (Fig. 3Ai and Supplementary Figure 1). Chloroquine (CQ) also inhibited ATP-induced IL-1beta secretion in THP-1 macrophages (Supplementary Figure 2). However, it was not able to inhibit nigericin-induced IL-1beta secretion (Fig. 3Aii). Western blot studies showed that HCQ treatment impaired the secretion of mature IL-1beta but did not modify the levels of pro-IL-1beta in the cell lysate when stimulated with ATP (Fig. 3B). Caspase 1 activation was also inhibited by HCQ in a dose-dependent manner. Again, HCQ could not inhibit IL-1beta nor caspase 1 activation triggered by nigericin (Fig. 3B). In agreement with our hypothesis, HCQ inhibited ATP (Fig. 3Ci) but not nigericin-induced K^+^ efflux in THP-1 macrophages (Fig. 3Cii). Our results from Fig. 3A–C suggest that HCQ does not seem to inhibit a process downstream the nigericin-triggered K^+^ efflux. This observation is in agreement with the fact that HCQ may inhibit endogenous K^+^ channels. We then induced inflammasome activation by promoting K^+^ efflux. We cultured cells in buffer without K^+^. As expected, this condition induced IL-1beta secretion. However, IL-1beta secretion was not inhibited by HCQ (Fig. 1D). Thus, HCQ might inhibit K^+^ channels that are actively regulated by other ion and/or second messenger. Along this line, HCQ did not impair cytosolic Ca^++^ increases induced by ATP (Supplementary Figure 3). These evidences support the hypothesis that HCQ controls inflammasome activation and that this effect is achieved through the inhibition of Ca^++^-activated K^+^ channels.

We then wished to determine whether HCQ inhibits inflammasome activation *in vivo*. To study this issue, we injected ATP i/p in WT and caspase 1–11^−/−^ mice. Intraperitoneal injection of ATP is known to recruit neutrophils in a caspase-1-dependent manner. HCQ treatment inhibited ATP-induced neutrophil recruitment, phenocopying the caspase 1–11^−/−^ mice (Fig. 1E). These results suggest that HCQ inhibits inflammasome activation *in vivo*.

Taken together, our results show that HCQ inhibits Ca^++^-activated K^+^ channels probably leading to impaired inflammasome activation.

## Discussion

Here we showed that HCQ can have anti-inflammatory properties through the inhibition of ion channels. This is a novel anti-inflammatory mechanism for antimalarials. Electrophysiology experiments directly showed that KCa1.1 and probably KCa3.1 channels are inhibited by HCQ. But which is the biological consequence of such inhibition? Our pharmacologic evidence suggests that these channels may be involved in inflammasome activation. We believe that the results showing that KCa1.1 and KCa3.1 activators promote IL-1beta secretion are not due to off-target effects. Those observations were in fact completely blocked by specific inhibitors of those channels. On the other hand, the fact that clotrimazole controls NS309-triggered IL-1beta also argues against off-targets effects of the inhibitor. Thus, the inhibition of Ca^++^-activated K^+^ channels by HCQ may lead to impaired inflammasome activation. We showed *in vitro* that HCQ inhibits ATP-induced caspase 1 activation and secretion of the mature form of IL-1beta in a dose dependent manner in human and mouse macrophages and dendritic cells. The following evidence support the interpretation that this effect is achieved through the inhibition of Ca^++^-activated K^+^ channels: i) HCQ inhibits ATP but not nigericin or low extracellular K^+^-induced inflammasome activation. ii) HCQ does not inhibit cytosolic Ca^++^. iii) HCQ impairs K^+^ efflux induced by ATP but not by nigericine.

We therefore believe that HCQ inhibits inflammasome activation *in vitro* and *in vivo*, probably through the inhibition of Ca^++^-activated K^+^ channels. Inhibited neutrophil recruitment obtained by HCQ treatment phenocopies caspase 1^−/−^ mice upon ATP injection (Fig. 3E). Within the context of our results shown in Figs 2 and 3A–D, our interpretation was that data on Fig. 3E could be explained because HCQ inhibits neutrophil recruitment through the inhibition of inflammasome activation. However, it is also possible that HCQ inhibits neutrophil recruitment through the control of migration by directly inhibiting KCa3.1 on those phagocytes^22^.

Ion channels are considered key determinants in the leukocyte biology. Among others, the Ca^++^-activated K^+^ channels KCa1.1 and KCa3.1 are believed to promote pathogenic inflammation. The discovery of inhibitors of these channels may be useful in chronic diseases. Along this line, recent papers have suggested that KCa1.1^8^ and KCa3.1^6, 7^ may play a pathogenic role in RA.

In SLE management, hydroxychloroquine (HCQ) is considered as the “gold standard” therapy^1^. Its withdrawn has been associated to increased lupus flares. Moreover, HCQ is believed to increase long-term survival of lupus patients. A key determinant of survival in SLE is cardiovascular disease. Carotid plaque is more prevalent and develops earlier in SLE patients as compared to healthy controls^23^. Along this line, HCQ improves the lipid and glucose metabolic profiles. We have shown that a short-term (3 months) treatment with HCQ led to decreased total cholesterol and LDL in SLE patients^24^. Metabolic syndrome has been associated to increased cardiovascular risk as well as cumulative organ damage in SLE patients^25^. Importantly, HCQ use might be a protective factor for cardiovascular disease in SLE^25^. Furtehrmore, NLRP3 inflammasome activation has been shown to play a key role in promoting atherosclerosis as well as type II diabetes^15^. Thus, inflammasome inhibition by HCQ might also explain the metabolic protective effects of the drug. Our results showing that HCQ inhibits inflammasome activation add a new mechanism by which HCQ can control autoimmunity and coronary disease.

In conclusion, our results show that HCQ inhibits Ca^++^-activated K^+^ channels. This effect may lead to impaired inflammasome activation. These results are the basis for i) a novel anti-inflammatory mechanism for HCQ and ii) a new strategy to target pro-rheumatic Ca^++^-activated K^+^ channels.

## Methods

### Whole cell patch-clamp

THP-1 macrophages were patched following standard procedures. Corning 7052 glass pipettes were pulled using a P-2000 micropette puller (Sutter Ins. Co.), to yield 3–5 MOhms pipettes filled with basic internal and external/bath solutions. The basic external/bath solution contained (mM): 140 NaCl, 5 KCl, 2 CaCl2, 1 MgCl2, 10 glucose, 10 Hepes (pH 7.4). The basic intracellular solution contained (in mM): 140 KCl, 10 NaCl, 1–2 EGTA, 3 Mg-ATP, 10 Hepes (pH 7.2); CaCl2 was added to bring free [Ca^++^] to the desired concentrations (100–300 nM), using maxchelator with affinity constants for Mg^2+^ and EGTA, obtained from Martell and Smith, 1977. Several K^+^ channels have been described in THP-1 cells. To get rid of IDR Kv channels, we applied 100 uM 4-aminopyridine (4-AP) to the bath. To get rid of IKr, we applied a P/12 protocol as control. Currents were recorded using a Model 2400 AM patch amplifier at 10 KHz, previously filtered at 2 KHz. (AM Systems Inc.), using pClamp (Molecular Devices). To evaluate a possible contribution of chloride currents, initial experiments were performed with Gluconate substitution by Chloride. No significant differences were obtained with the Chloride containing solutions (data not shown). Data was analyzed using Clampfit (Molecular Devices) and Sigmaplot 11 (Jandel Scientific). When evaluating a possible role of HCQ in the remaining Ca^++^ activated K^+^ currents, HCQ was added to the bath at a concentration range varying between 1–30 µM.

### Cell lines and primary cultures

Mouse BMDC and human monocyte-derived DCs were generated *in vitro* as previously described^26^. THP-1 cells (ATCC^®^ TIB-202™) were differentiated to macrophages by treating them with 0.1 µM PMA during 48 hs.

### Reagents

HCQ, LPS, nigericin, ATP, NS169, NS309, clotrimazol, IBTX, TEA and BAPTA-AM were from Sigma (St Louis). Anti-IL-1beta and anti-caspase 1 were from Santa Cruz Biotechnologies. Human IL-1beta ELISAs were from BD.

### *In vivo* inflammasome activation

Animal experimentation protocol (004–15) was approved by the Animal Ethics Committee from Institut Pasteur de Montevideo. All methods were performed in accordance with the relevant guidelines and regulations. 6–10 weeks old C57BL6/NJ WT and caspase 1–11^−/−^ from Jackson Laboratories were used. 20 mg/kg ATP was injected i/p to saline or HCQ (1 mg/kg) i/p treated animals. HCQ was done daily since day - 7. 4 hs after ATP injection, peritoneal lavage was done. Neutrophils were quantified by FACS using anti-CD11b and anti-Ly6G antibodies.

### Flow cytometry

Peritoneal lavage was recovered and cells were stained with anti-CD11b-APC (BD, clone M1710, dilution 1/500), anti-Ly6G-PE (BD, clone 1A8, dilution 1/200) and anti-Ly6C-FITC (BD, clone AL-21, dilution 1/200) for 20 minutes at 4 °C in PBS-0.2% SBF-0.1% sodium azide (PSA). Cells were wash 2 times with PSA and then analyzed using BD Accuri C6 (BD Bioscience) or CyAn ADP (Beckman Coulter) flow cytometer. Data analysis was performed with FlowJo software. Between 8 and 5 million cells were recovered from the peritoneal lavage, and at least 20000 single cell events were recorded.

### Statistical analysis

One-way anova analysis was performed to compare three or more groups using Tukey’s comparison.

## Electronic supplementary material

Supplemental Figures
