# Supplementary material for: Pro-inflammatory Ca++-activated K+ channels are inhibited by hydroxychloroquine
Source: Sci Rep. 2017 May 15;7:1892. doi: 10.1038/s41598-017-01836-8 (PMC5432501; doi:10.1038/s41598-017-01836-8)

Supplementary Figure 1. HCQ inhibits ATP-induced IL-1beta secretion

Human monocyte-derived DCs (A), mouse BMDCs (B) and murine immortalized macrophages (C) were treated with the indicated compounds.

In all the cases LPS was used at 0,25mg/ml during 3 hours. Cells were washed and then ATP was added at 2 mM (DCs) or 5mM murine macrophages for 45 minutes. When indicated, HCQ was added during ATP treatment at 3 (+) or 30 µM (++).

One experiment representative of at least three is shown. *=p<0,05; **=p<0,01; ***=p<0,001

A

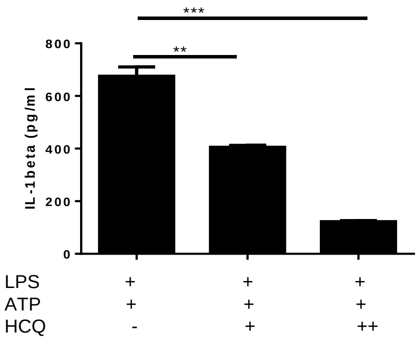

B

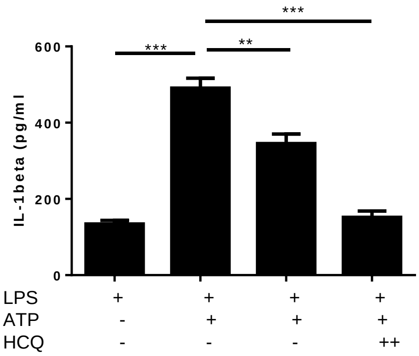

C

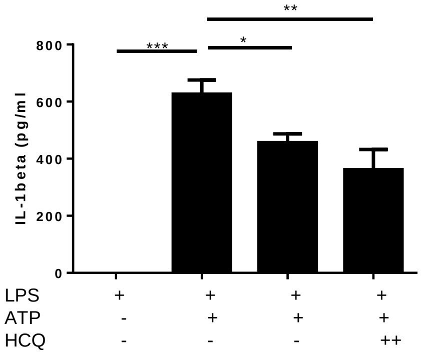

Supplementary Figure 2. Chloroquine (CQ) inhibits ATP-induced IL-1beta secretion

THP-1 macrophages were treated with LPS at 0,25mg/ml during 3 hours. Cells were washed and then ATP was added at 5mM for 45 minutes. When indicated, CQ was added during ATP treatment at 1 (+) or 10 µM (++).

One experiment representative of three is shown. *=p<0,05; **=p<0,01;

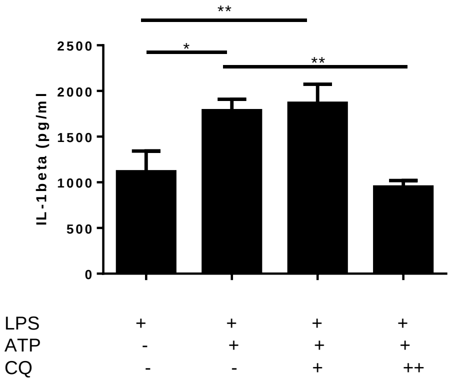

Supplementary Figure 3. HCQ does not inhibit ATP-induced liberation of cytosolic Ca^{++}.
Cells were loaded with Fura-2. 340/380 emission fluorescence was registered through videomicropscopy. At "time 0" 5 mMATP was added to the culture. Cells were left untreated or treated with 10µM HCQ 30 minutes before the registration. One experiment representative of three is shown.

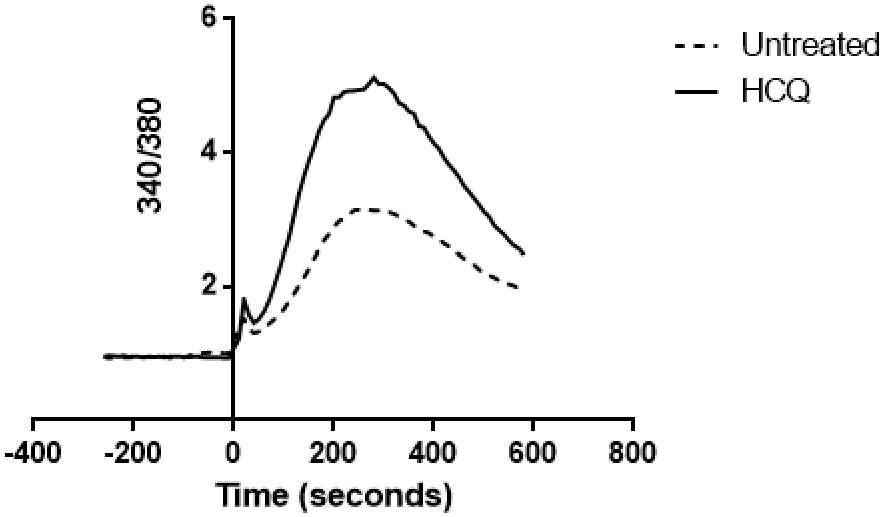

Supplement: Supplementary file 1 — Supplemental Figures [file 41598_2017_1836_MOESM1_ESM.pdf]
